# Implementing video games to enhance the surgical skills among oral cancer surgeons

**DOI:** 10.1097/JS9.0000000000000111

**Published:** 2023-03-02

**Authors:** Saravanan Sekaran, Priyadharshini Ranganathan, Suresh Kumar Rajamani Sekar

**Affiliations:** Departments of a Prosthodontics; b Oral Pathology, Saveetha Dental College and Hospitals, Saveetha Institute for Medical and Technical Sciences, Chennai, Tamil Nadu, India; c College of Natural and Computational Sciences Arba Minch University, Ethiopia

*Dear Editor*,

Recently, we have read the correspondence entitled “How video games can help medical students become better surgeons in the future – correspondence” by Muhammad *et al* 1. We congratulate the authors for an interesting article which provides insights to incorporate video games in the medical curriculum. We take the liberty of adding our thoughts of implementing video games to improve the surgical skills of oral cancer surgeons.

Learning video games has gained popularity as a method for medical education, health promotion, and illness prevention due to their enjoyable and engaging features including competition and feedback elements. The surgical healthcare workers were tested and trained using digital video game–based learning. In addition to their clinical hours, trainee surgeons can develop their surgical abilities outside operating rooms by playing video games. Since then, playing video games has been a successful way to trade surgeons^2^.

For aspiring surgeons to minimize errors and expedite surgery, nontechnical skills including excellent communication skills, teamwork, leadership, attention, and decision-making are also essential. Playing video games facilitates the blending of challenges and learning. It guarantees achieving the main aim in stealth mode learning, and players are encouraged to play until they succeed^3^. Suliman *et al*.^4^ pointed out the advantages of multiplayer video games for improving decision-making, speed, decreasing errors, improving problem-solving skills, increasing the proper utilization of both human and instrumental resources with a comfortable and adequate work environment, and speeding up the completion of the procedure. Hand-eye coordination, visual spatial ability, and depth perception can all be enhanced with the help of surgical training and video games. The advantages of video games as a supplemental method to enhance surgical abilities in robotic and laparoscopic procedures is through enhancing brain plasticity and which aids to modify the baseline of invasive surgical techniques that were used in laparoscopic surgery after a minimally invasive treatment^5^. A brief study was conducted on a realistic laparoscopic course for surgical training using a smartphone camera and a handcrafted wooden simulator box. The findings revealed that people with prior gaming experience needed less time to complete the laparoscopic stitching in the simulator box^6^. The surgical trainee’s performance in difficult procedures, such as knot tying, minimally invasive surgical skill environments, and camera navigation in laparoscopic surgery, was improved by COVID-19 virtual reality simulator training^7^.

For surgical oncology, laparoscopic suturing, and the use of ocular lasers, it is also essential to have appropriate vision and focusing abilities to prevent over sectioning of the operative margins. Green and Bavelier^8^, found that video games can help people to get better at handling stressful scenarios and situations that require hand-eye coordination, depth perception, and visuospatial abilities. In a cross-sectional survey of 33 medical residents, James C. Rosser Jr. *et al.* 9 examined the effectiveness of past and present experience with gun drill video games versus suturing and laparoscopic skills after surgery. They found that 32% of current video game players made few mistakes, while 37% of past video game players made mistakes. In response to questions on faster performance, 24% of former video gamers and 27% of current video gamers demonstrated quick performance.

Facial disfigurement following surgery in oral cancer patients may often prove to be socially stigmatizing and predisposing patients to mental health disorders. Reconstructive surgery is a key to the management, its quality determines patient survival and morbidity. Microsurgical reconstruction of the oral and maxillofacial area is a challenging procedure which requires unique motor skills and coordinated hand-eye movement among the surgeons. Despite modernized and advanced surgical tools, surgical outcome is greatly influenced by the surgical skills. A well-defined preoperative plan by the oncosurgeon is essential and governs the postoperative success of the oncological procedure. Better abilities in assessing and sectioning the primary tumor invaded borders together with normal surrounding tissue, followed by suturing, are especially needed in oncosurgery. Since gaming can enhance dexterity and surgical procedural skills and has a great impact on curriculum-based learning, it must be included in the curriculum for training oral cancer surgeons.

## Ethical approval

Not applicable.

## Sources of funding

None.

## Authors’ contribution

S.S., P.R., and S.K.R.S. devised the concept, performed the literature search, and drafted the letter.

## Conflicts of interest disclosure

The authors declare that they have no financial conflict of interest with regard to the content of this report.

## Research registration unique identifying number (UIN)

None.

## Guarantor

Not applicable.
